# Breastfeeding and the 2015 South African guidelines for prevention of mother-to-child transmission of HIV

**DOI:** 10.4102/sajhivmed.v16i1.377

**Published:** 2015-05-20

**Authors:** Louise Kuhn, Max Kroon

**Affiliations:** 1 Gertrude H. Sergievsky Center, College of Physicians and Surgeons, Columbia University, United States; 2 Department of Epidemiology, Mailman School of Public Health, Columbia University, United States; 3 Division of Neonatal Medicine, Department of Paediatrics, University of Cape Town, South Africa

Breastfeeding, especially exclusive breastfeeding in the first months of life, is the cornerstone of good infant nutrition, health and survival. The various health benefits include the mother and also extend beyond infancy to protection against common noncommunicable diseases in adult life. These benefits take on even greater salience in low-resource settings.

Mother-to-child transmission (MTCT) of HIV through breastfeeding and the Centers for Disease Control's initial recommendation that HIV-infected women avoid breastfeeding their infants, threatened this key child health-promoting activity. Support for breastfeeding by HIV-infected women is steadily being reinstated, however. This change was prompted by numerous reports that formula feeding incurred significant harm^1^ and was also facilitated by a 1999 report that showed significantly reduced postnatal HIV transmission if breastfeeding was exclusive.^2^ Further impetus came from a demonstration that postnatal transmission is almost eliminated if maternal combination antiretroviral treatment (cART) or extended infant antiretroviral peri-exposure prophylaxis (PEEP) is provided during breastfeeding.^3^ In 2012, even the British HIV Association (BHIVA) guidelines permitted breastfeeding under tightly controlled circumstances, if mothers insisted.

The 2010 South African Prevention of Mother-To-Child HIV Transmission (PMTCT) Guidelines incorporated this evidence and were broadly supportive of HIV-infected women breastfeeding their infants, but stopped short of adopting breastfeeding as the programme's default feeding choice. The Tshwane Declaration in August 2011 shifted South Africa squarely onto the breastfeeding restoration path. Besides promoting, protecting and supporting breastfeeding generally, the declaration specifically adopts breastfeeding as the default feeding method for HIV-exposed infants and promotes human milk banks to support breastfeeding and breast milk feeding. The Tshwane Declaration was followed by the promulgation of regulations (R991/2012) to enforce the international code on marketing of breast milk substitutes. Nevertheless, South Africa still lags behind many other African countries in the uptake and duration of breastfeeding and the duration of exclusive breastfeeding.^4^

New guidelines to improve PMTCT in South Africa have recently been released and recommend lifelong cART for all pregnant women and those who have delivered in the preceding 12 months, irrespective of disease and laboratory criteria.

Four important new dimensions provide greater attention to neonates, namely (1) birth polymerase chain reaction (PCR) testing of high-risk neonates, (2) extended infant antiretroviral (ARV) prophylaxis if early *breastfeeding risk is increased* owing to inadequate duration of maternal cART, (3) combination infant ARV prophylaxis (cARP) if *intrapartum transmission risk is increased* and (4) very early initiation of cART in infected neonates. In the present article, we discuss the implications of these new programme and policy developments for breastfeeding.

The first issue is the specific counselling needs of women whose infants are HIV-infected. The new birth PCR testing programmes will identify most HIV-infected infants much earlier than the old 6-weeks PCR testing programmes.^5^ This approach provides a valuable opportunity for strengthening support for breastfeeding, which is essential to the survival and well-being of HIV-infected infants. HIV-infected infants should be breastfed for 2 years or more.

Secondly, by design, the new, targeted birth testing programmes will encompass a large proportion of preterm and low birthweight (LBW) neonates. Many challenges exist to maintain optimal breastfeeding and clinical care for this high-risk group, regardless of HIV exposure status. HIV clinicians will need to improve their skills and expertise in this area as caring for more of these vulnerable infants becomes their responsibility. In turn, neonatologists and paediatricians already caring for this clinical population will need to strengthen their skills and expertise in HIV-related interventions.

Thirdly, *risk estimation* linked to targeted testing or augmented infant prophylaxis will identify women with suboptimal cART, including those who have not yet accessed care, those who deliberately avoided care, those who are non-adherent, those who are failing cART, late starters, etc. Counselling around infant feeding for this group will need to balance HIV transmission risks against the adverse and potentially fatal outcomes associated with abstinence from breastfeeding, particularly in preterm and LBW infants.

## HIV-infected neonates

Infant feeding counselling in PMTCT programmes has mostly ignored the scenario of known HIV-infected neonates. Typically, counselling has been directed at HIV-infected women with infants of unknown HIV status. Whilst antenatal counselling still has to keep this focus, postnatal counselling will now have the benefit of much earlier diagnosis of HIV infection than was previously possible. If a sample for a PCR test is collected at birth, the mother should be able to learn her infant's HIV status in a matter of days, depending on the turnaround time of the laboratory and the schedule of follow-up visits. This time could be reduced to a few hours if point-of-care (POC) tests are used routinely in the clinical setting. For an infant diagnosed as infected, earlier diagnosis offers a valuable opportunity for strengthening support for breastfeeding. Breastfeeding is crucial for the wellbeing of HIV-infected infants who are at exceedingly high risk of mortality.^6^ Breastfeeding for two years or more can be unequivocally recommended and supported for the infected infant, as the risk of HIV transmission is no longer a consideration.

However, learning the infant's diagnosis is challenging for mothers. Many are distressed and experience feelings of guilt. They should be reassured that any ‘re-infection’, should it even occur, will not accelerate disease progression in their infant and that the protective benefits of breastfeeding in an HIV-infected child far outweigh the minuscule possibility of harm.

Very early diagnosis also provides a window of opportunity to begin or restart breastfeeding for those who either decided to forgo all breastfeeding or who stopped before the infant's diagnosis. Healthcare workers and the community are often quite ignorant of the reversible nature of infant feeding decisions. An innovative programme in Soweto demonstrated that a modest relactation counselling program had reasonable success in achieving full lactation after infant HIV diagnosis, even in mothers who had abstained entirely from breastfeeding until infant diagnosis around 12 weeks of age.^7^

## Preterm and low birthweight neonates

Preterm and LBW infants are generally at higher risk of perinatal HIV transmission than term, normal birthweight infants. They also may have biological reasons for increased susceptibility to enteral acquisition of HIV from breast milk. In addition, preterm birth before adequate passive transfer of maternal neutralising antibodies may cause reduced protection against postnatal HIV infection. This is why preterm birth and/or LBW is included as one of the criteria for targeted birth testing and, in the Western Cape, for cARP.

Few studies of extended infant prophylaxis include preterm infants, despite their higher risk of infection, and special dosage considerations are required in this group.^8^ Nevertheless, combining infant PEEP with maternal cART is advisable in this group, as avoidance of breastmilk feeding increases morbidity and mortality significantly.

The immature, preterm gut is nowhere near as robust as the term gut with, initially, relative hypomotility, underdeveloped microvilli and brush border enzymes predisposing to malabsorption, stasis, bacterial overgrowth and inflammation, particularly if fed injudiciously. Very gentle graduated feeding with human milk reduces the risk of intestinal inflammation and necrotising enterocolitis (NEC). NEC is three times more common with formula milk than with donated human milk feeding.^9^ Gut inflammation is likely to increase the number of CD4 cells and CCR5 receptor expression and increase vulnerability to HIV transmission. Additionally, HIV-exposed infants may be more at risk of developing NEC, worse grades of progressive NEC and mortality from NEC with worse outcomes after surgery.^10^

Consequently, whilst human milk feeding is critical to better outcomes in HIV-exposed preterm neonates, it may involve the risk of HIV transmission despite ARV prophylaxis. It may be advisable to complement infant PEEP and maternal cART to further reduce transmission risk. Consideration should be given to heat treatment of the infant's own mother's milk or feeding with human milk from an HIV-negative donor at least temporarily whilst feeds are being established and preterm gut matures in the first few weeks of life. Over-reliance on donated human milk should be discouraged, as this does not facilitate sustained breastfeeding after discharge whilst heat treatment of own mother's milk does.

Human milk banks have a critical role in supporting breastmilk feeding, and the South African Department of Health is currently developing regulations on milk banking. Some provinces and non-governmental organisation (NGOs) have already developed milk banks and milk bank networks.

One of the postulated mechanisms for the increased rate of transmission in mixed breastfeeding infants is that cow's milk protein or solid food causes low-grade gut inflammation, thus increasing susceptibility. This possibility may be especially true for the immature gut. Preservation of exclusive breastmilk feeding may be facilitated by the temporary use of donated human milk until lactation is fully established or the next batch of own mother's milk is brought from home.

There are some data to suggest that heat treatment of expressed breast milk (EBM) at home may be implementable but this complicated approach requires a great deal of motivation from family and adequate support from the health service. The approach is worth considering in preterm neonates *in hospital* with additional risk factors such as mothers who fail therapy or who are drug resistant. The bulk of feeds would initially be by gastric tube, and this facilitates heat treatment of EBM. Cup feeding of heat-treated EBM may also be considered. It is probably safe to transition to suckling directly from the breast with extended infant ARP and maternal cART cover once the gut has matured and full enteral feeds are established and well tolerated. Minimally nutritive suckling may accelerate oro-motor maturation and should be encouraged.

Sustaining lactation in mothers of preterm infants can be challenging, particularly when faced with meagre lodging facilities, prolonged maternal-infant separation especially because of severe maternal illness, infrequent visiting owing to poverty or substance abuse, and inadequate support for sibling care especially in recently migrated impoverished families. In addition, postnatal depression and poor advice from healthcare workers may undermine sustained breastfeeding. Mothers should be informed that fortification of their milk to meet the increased nutrient demands of the preterm infant is preferable to special preterm formula. Whilst some infrastructure issues may be dealt with at a health systems level, commitment to the Mother and Baby Friendly Initiative (MBFI) principles, Kangaroo Infant Care and promotion of routine early and regular emptying of breasts by manual and mechanical expression are vital to support and sustain breastfeeding. Pharmacological interventions to optimise milk expression may also be helpful but, by and large, the most important component is parental education and ‘buy-in’ of the benefits of breastfeeding. An institutionalised belief that breastfeeding is a critical component of preterm care goes a long way to reverse the tendency to rely on formula milk as a short-term, quick-fix option.

This complex subset of preterm and LBW infants neonates poses many challenges for ensuring optimal and sustained breastfeeding and care whilst preventing HIV infection. HIV clinicians, neonatologists and paediatricians will have to rise to this challenge as these babies become the focus of intensified risk-based prophylaxis and, if infected, cART in the first weeks of life.

Some hospitals have sophisticated programmes to help support breastfeeding of preterm infants including kangaroo infant care, heat-treatment stations and active milk banks. Other hospitals, however, need to establish this capacity as soon as possible.

## Neonates of mothers who are non-adherent or have drug-resistant virus

Infants whose mothers have had suboptimal ARV exposures will constitute the majority of high-risk infants identified for targeted testing or cARP. This group includes women who have only recently learned their HIV status; those who have deliberately absented themselves from programmes, are non-adherent or defaulters and those not yet able to access appropriate services. All of these characteristics point to challenging social circumstances. Ensuring that the mothers of these high-risk infants obtain HIV-related care and ARVs necessary for their own health is as important as ensuring the best available prophylaxis for their infants.

Given the probable social disadvantage of women who are partially adherent to cART, the new advice that women who are failing second- or third-line treatment should formula feed is, in most circumstances, ill-advised. There are no special reasons to avoid breastfeeding in this group. The same risk/benefit considerations for breastfeeding in all HIV-exposed infants apply to this subgroup, and many will have social circumstances that could amplify the adverse consequences of avoiding breastfeeding.

Invoking risk of transmission of drug-resistant virus through breastfeeding as the motivation for avoiding it is not logical. Concerns about transmission of drug resistance in this situation are overstated and confused. Almost all infants who fail PMTCT (i.e. become infected despite being exposed to ARVs) have virus resistance to a number of first-line ARVs.^11^ The paediatric treatment guidelines already address this by recommending initiation with ritonavir-boosted lopinavir (LPV/r)-based cART for HIV-infected infants from 42 weeks’ corrected gestational age and young children under 3 years of age. MTCT of resistance to LPV/r is exceedingly rare. We are aware of one report of transmitted LPV/r resistance to an infant via perinatal rather than postnatal transmission.^12^ Trials using LPV/r for PMTCT have not observed frequent emergence of LPV/r resistance in either mothers or infants.^13^ Even if it were to occur, the small risk of resistance applies only to the small population of infants who are infected. Avoiding breastfeeding for the benefit of a tiny minority places the majority of HIV-exposed uninfected infants at risk of poor health and development.

**FIGURE 1:**
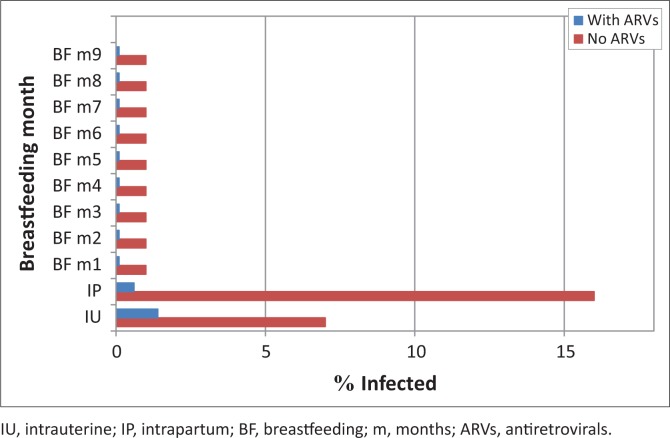
Risks of HIV transmission with (in red) and without (in purple) antiretroviral drugs occurring during the intrauterine, intrapartum and during months 1–9 via breastfeeding. The risks of transmission amongst mothers who are non-adherent, failing therapy or drug resistant are likely to lie between these two estimates.

Fear of HIV transmission during breastfeeding looms large, and fear of transmission of drug-resistant HIV even larger. This fear seems to blind providers to the immediate risks of poor growth, pneumonia and diarrhoea, significantly more likely to be worse or fatal in the non-breastfed infant.^1^ The risks of postnatal HIV transmission are almost 20 times less than during the pregnancy and delivery. ARVs given to the mother or to the infant reduce risks via all of these routes by a factor of more than 10 (Figure 1). In the event of suboptimal adherence to ARVs, the risk of HIV transmission does not exceed that observed in the absence of ARVs. Risks of transmission in this partially adherent group will be less than the risks when no ARVs are given.

The social circumstances and health service access issues associated with this subgroup tend to exacerbate the adverse effects of abstinence from breastfeeding. Formula feeding should only be considered in this group if all options of ensuring access to ARVs for mother and infant have been exhausted or there are absolutely no prospects of breastfeeding because serious maternal substance addiction will grossly interfere with it.

## Conclusion

Risk recognition linked to improved testing and infant prophylaxis may further reduce transmission, diagnose infection earlier and improve linkage to definitive care and treatment. There is a danger that when transmission risk is increased, replacement feeding may be considered to prevent postnatal transmission despite breastfeeding being critical to infant health and survival. The few paediatric infections averted will be at the expense of harm to the majority who are HIV-exposed but uninfected.

Even with imperfect prophylaxis, less than 1% of infants become infected in each month of breastfeeding. Promoting replacement feeding denies breastfeeding benefits to the more than 99% of HIV-exposed infants who remain uninfected. Importantly, these benefits would also be denied to the high-risk infants infected during birth and only diagnosed at 6 weeks of age or older. Clinicians may struggle to keep this in mind when counselling the individual ‘high-risk’ patient on feeding choices, and should avoid inflating the real but tiny risk of transmission and understating the real harm from not breastfeeding.

At a public health level, occasional individual infections via breastfeeding are a small price to pay for a compelling benefit to the majority. The low frequency of transmission during breastfeeding, even when risk is increased, allows time to optimise maternal cART to reduce risk rather than promoting formula feeding.

A weak point in the old early infant diagnosis programme was a tendency towards early termination of breastfeeding around 10–12 weeks when receiving the negative result from the 6-weeks test. This pattern suggested a missed opportunity to support breastfeeding at this critical juncture. It is unclear whether counselling specifically encouraged women to stop breastfeeding or whether messaging around the need for retesting inadvertently failed to convey the importance of continued breastfeeding. Vigilance is needed when counselling mothers about the meaning of negative birth PCR tests to ensure that this counselling does not inadvertently discourage breastfeeding.

Breastfeeding and breastmilk feeding remain the best feeding method to optimise health outcomes in PMTCT, even when mothers are failing first- and second-line treatment. The counselling messages after birth testing must include clear support for breastfeeding if we are to leverage the full health benefits of very early HIV diagnostic testing and strengthened prophylaxis.
